# Supplementary material for: Gabapentinoids confer survival benefit in human glioblastoma
Source: Nat Commun. 2025 May 15;16:4483. doi: 10.1038/s41467-025-59614-4 (PMC12081740; doi:10.1038/s41467-025-59614-4)
Supplement: Supplementary file 1 — Supplementary Information [file 41467_2025_59614_MOESM1_ESM.pdf]

SUPPLEMENTARY INFORMATION

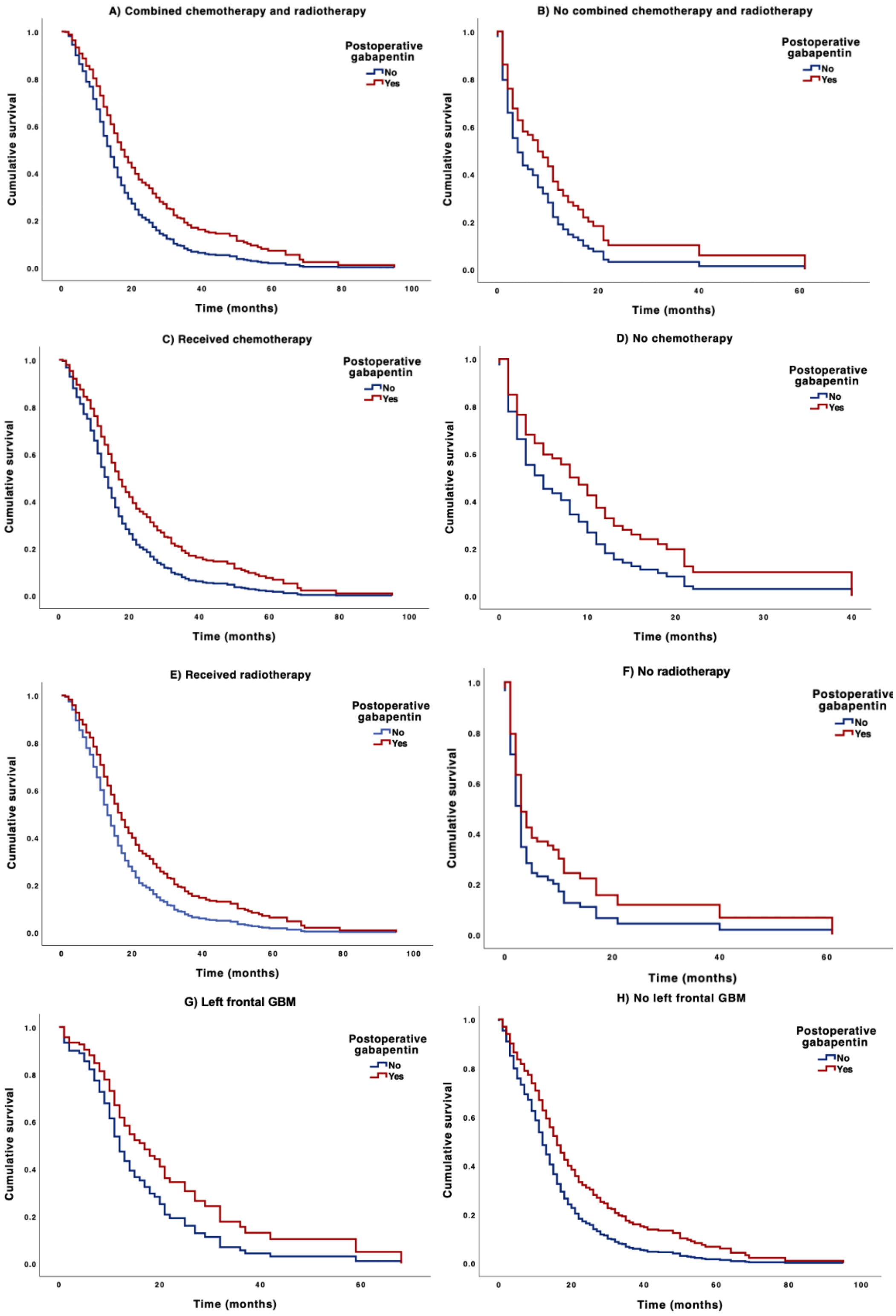

Supplementary Figure 1. **Stratified survival analyses.** Stratified Cox plots of overall survival following initial surgical resection comparing patients who received gabapentin (red) to those who did not (blue). Including chemotherapy and radiotherapy (**A-F)** as additional covariates confirmed the overall survival benefit associated with gabapentin (HR = 0.72; 95%CI = 0.56-0.92). Likewise, including left-frontal tumor (**G-H**) as an additional covariate confirmed the overall survival benefit associated with postoperative gabapentin (HR = 0.66; 95%CI = 0.51-0.84).

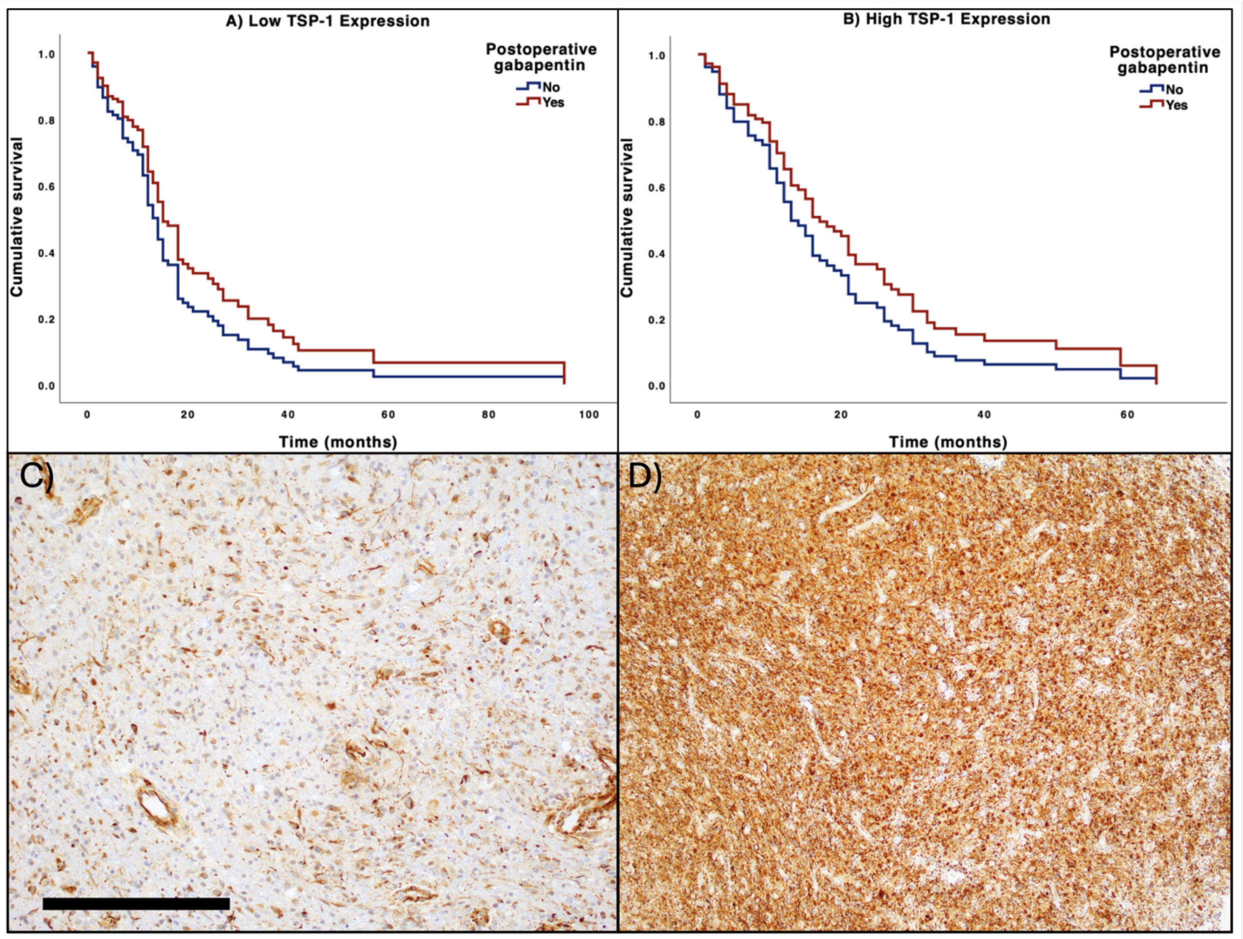

Supplementary Figure 2. **Stratified TSP-1 analysis by low (A) and high (B) TSP-1.** 44 patients with low TSP-1 expression received gabapentin, 55 patients with low TSP-1 expression did not receive gabapentin. 51 patients with high TSP-1 expression received gabapentin, 41 patients with high TSP-1 expression did not receive gabapentin. Cox plots for overall survival following initial surgical resection comparing patients who received gabapentin to those who did not; levels of TSP1 were not related to response to gabapentin. Example of low TSP-1 (**C**) (<50% of cells) and high TSP-1 (**D**) (>50% of cells) staining. The scale bar represents 100 μM.

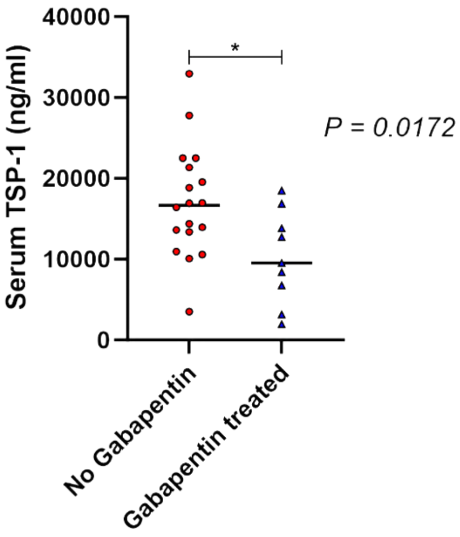

Supplementary Figure 3: Serum TSP-1 quantification following initiation of gabapentin. Serum TSP-1 quantified by ELISA for the gabapentin patients (n = 9; blue) and no gabapentin controls (n = 18; red) matched by age, tumor volume and EOR. Mean serum TSP-1 in gabapentin patients was significantly lower when compared with untreated controls (10,181 ng/ml vs 17,015 ng/ml, p=0.017).

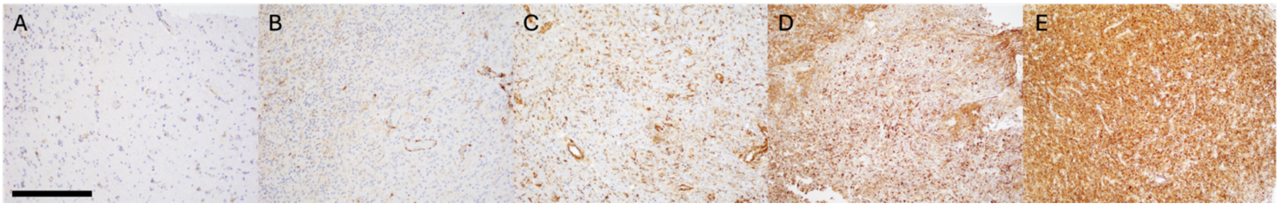

Supplementary Figure 4. **Expression patterns for different scores of TSP-1**. <5% stained (**A**), 5-25% stained (**B**), 25-50% stained (**C**), 50-75% stained (**D**) and >75% stained (**E**). Scale bar represents 50 µM for (A) and (B) and 100 µM for (C), (D), and (E).

Supplementary Table 1. Patient characteristics of the discovery and validation cohorts.

	Discovery Cohort (MGB)			Validation Cohort (UCSF)		
Variable	**Post-operative Gabapentin (n = 103)**	**No Post-Operative Gabapentin (n = 590)**	**Total (n = 693)**	**Post-operative Gabapentin (n = 36)**	**No Post-Operative Gabapentin (n = 343)**	**Total (n = 379)**
Age at Diagnosis, Years, Median (IQR)	63 (56 – 72)	65 (57 – 73)	65 (57 – 73)	58.1 (50.1 – 65.8)	61.4 (53.7 – 68.2)	60.9 (53.0 – 67.7)
Sex (n, %)						
Females	55 (53.4%)	263 (44.6%)	318 (45.9%)	18 (50.0%)	128 (37.3%)	146 (38.5 %)
Males	48 (46.6%)	327 (55.4%)	375 (54.1%)	18 (50.0%)	215 (62.7%)	223 (61.5%)
Race (n, %)						
White	90 (87.4%)	539 (91.4%)	629 (90.8%)	34 (94.4%)	311 (91.0%)	345 (91.5%)
Black / African American	2 (1.9%)	14 (2.4%)	16 (2.3%)	2 (5.6%)	19 (5.6%)	21 (5.6%)
Asian / Pacific islander	5 (4.9%)	6 (0.9%)	11 (1.5%)	0 (0.0%)	6 (1.8%)	6 (1.6%)
Other	6 (5.8%)	31 (5.3%)	37 (5.3%)	0 (0.0%)	5 (1.5%)	5 (1.3%)
Ethnicity (n, %)						
Hispanic	2 (1.9%)	13 (2.2%)	15 (2.2%)	2 (5.6%)	11 (3.2%)	13 (3.5%)
Non-Hispanic	101 (98.1%)	577 (97.8%)	678 (97.8%)	34 (94.4%)	325 (95.3%)	359 (95.2%)
Unknown	0	0	0	0 (0.0%)	5 (1.5%)	5 (1.3%)
Tumor Laterality (n, %)						
Left	51 (49.5%)	275 (46.6%)	326 (47%)	18 (50.0%)	171 (49.9%)	189 (49.9%)
Right	52 (50.5%)	310 (52.5%)	362 (52.2%)	18 (50.0%)	165 (48.1%)	183 (48.3%)

Bilateral	0	5 (0.9%)	5 (0.8%)	0 (0.0%)	2 (0.6%)	2 (0.5%)
Unknown	0	0	0	0 (0.0%)	5 (1.5%)	5 (1.3%)
Seizure preoperatively, (n, %)						
Yes	21 (20.4%)	101 (17.1%)	122 (17.6%)	23 (63.9%)	172 (50.2%)	195 (51.5%)
No	82 (79.6%)	489 (82.9%)	571 (82.4%)	13 (36.1%)	171 (49.9%)	184 (48.6%)
Baseline KPS, (n, %)						
≤80	6 (5.8%)	39 (6.6%)	46 (6.6%)	12 (33.3%)	106 (30.9%)	118 (31.1%)
90	5 (4.9%)	26 (4.4%)	31 (4.5%)	7 (19.4%)	79 (23.0%)	86 (22.7%)
100	1 (0.9%)	9 (1.5%)	10 (1.4%)	3 (8.3%)	13 (3.8%)	16 (4.2%)
Unknown	91 (88.4%)	516 (87.5%)	606 (87.5%)	14 (38.9%)	145 (42.3%)	159 (42.0%)
MGMT status, (n, %)						
Unmethylated	54 (52.4%)	328 (55.6%)	384 (55.4%)	10 (27.8%)	72 (21.0%)	82 (21.6%)
Methylated	47 (45.6%)	234 (39.7%)	281 (40.5%)	6 (16.7%)	83 (24.2%)	89 (23.5%)
Unknown	2 (1.9%)	28 (4.7%)	28 (4.1%)	20 (55.6%)	188 (54.8%)	208 (54.9%)
Adjuvant chemoradiation (n, %)						
Both	84 (81.6%)	464 (78.6%)	548 (79.1%)	29 (80.6%)	283 (82.5%)	312 (82.3%)
Radiation only	10 (9.7%)	42 (7.1%)	52 (7.5%)	2 (5.6%)	18 (5.3%)	20 (5.3%)
Chemotherapy only	2 (1.9%)	20 (3.4%)	22 (3.2%)	2 (5.6%)	15 (4.4%)	17 (4.5%)
Neither	7 (6.8%)	64 (10.8%)	71 (10.2%)	3 (8.3%)	22 (6.4%)	25 (6.6%)
Unknown	0	0	0	0 (0.0%)	5 (1.5%)	5 (1.3%)
Extent of resection, (n, %)						

Sub-total resection	38 (36.9%)	289 (49%)	327 (47.2%)	-	-	-
Gross-total resection	59 (57.3%)	264 (44.7%)	323 (46.6%)	-	-	-
Biopsy	6 (5.8%)	35 (5.9%)	41 (5.9%)	-	-	-
Unspecified	0 (0%)	2 (0.4%)	2 (0.3%)	-	-	-
Volume of residual tumor following resection, mL, No. (%)						
<25	-	-	-	24 (66.7%)	164 (47.8%)	188 (49.6%)
25-49	-	-	-	4 (11.1%)	101 (29.5%)	105 (27.7%)
50-99	-	-	-	7 (19.4%)	61 (17.8%)	68 (17.9%)
100-300	-	-	-	1 (2.8%)	15 (4.4%)	16 (4.2%)
Unknown	-	-	-	0 (0.0%)	2 (0.6%)	2 (0.5%)
Volume of residual tumor following resection (IQR), mL	-	-	-	15.8 (5.6 - 46.2)	26.7 (10.5 - 48.5)	25.0 (9.7 - 48.3)

MGB = Mass General Brigham; UCSF = University of California San Fransisco; IQR = Interquartile range.

Supplementary Table 2: Gabapentin treatment dosing, timing, concurrent chemotherapy/radiotherapy, and survival in GBM patients within the MGB discovery cohort.

Patient Sequence	Duration of Gabapentin (in days)	Weighted Average Daily Dosage (in mg)	Preoperative Gabapentin	Postoperative Radiotherapy	Postoperative Chemotherapy	Postoperative Survival (in months)
Patient 1	148	300	No	Yes	Yes	95
Patient 2	111	1354	No	Yes	Yes	14
Patient 3	335	920	No	Yes	Yes	11
Patient 4	Unknown	600	Yes	Yes	Yes	18
Patient 5	82	900	Unknown	Yes	Yes	5
Patient 6	Unknown	600	Yes	Yes	Yes	4
Patient 7	Unknown	300	Yes	Yes	Yes	32
Patient 8	40	300	No	Yes	Yes	28
Patient 9	808	1526	No	Yes	Yes	50
Patient 10	11	200	Yes	Yes	Yes	5
Patient 11	26	1800	Unknown	Yes	Yes	25
Patient 12	1605	300	Unknown	Yes	Yes	16
Patient 13	539	300	Yes	Yes	Yes	32
Patient 14	141	600	Unknown	Yes	Yes	12
Patient 15	Unknown	Unknown	Unknown	Yes	Yes	14
Patient 16	921	576	Unknown	Yes	Yes	34
Patient 17	53	1200	Unknown	Yes	Yes	39
Patient 18	240	900	Unknown	Yes	Yes	9
Patient 19	1090	882.6	Yes	Yes	Yes	37
Patient 20	67	636	No	Yes	Yes	18
Patient 21	664	1200	Yes	Yes	Yes	4
Patient 22	86	1794	No	Yes	Yes	13
Patient 23	258	900	Yes	No	Yes	4
Patient 24	40	818	Yes	No	No	2
Patient 25	159	300	No	Yes	Yes	10
Patient 26	Unknown	300	No	Yes	Yes	Alive
Patient 27	202	1800	No	Yes	Yes	13
Patient 28	1	200	No	Yes	Yes	Alive
Patient 29	1	300	No	No	No	3
Patient 30	38	1642	No	Yes	Yes	42
Patient 31	74	900	Yes	Yes	Yes	9
Patient 32	348	190	No	Yes	Yes	21
Patient 33	46	600	No	Yes	Yes	19
Patient 34	413	235	Yes	Yes	Yes	4
Patient 35	2	600	No	Yes	Yes	30
Patient 36	70	100	No	Yes	Yes	30
Patient 37	1604	2297	Yes	Yes	Yes	2
Patient 38	Unknown	900	Yes	Yes	Yes	26
Patient 39	796	434	Yes	Yes	Yes	27
Patient 40	176	900	No	Yes	Yes	Alive

Patient 41	21	100	No	Yes	Yes	Alive
Patient 42	1215	600	Yes	No	No	Alive
Patient 43	Unknown	300	No	Yes	Yes	14
Patient 44	9	900	No	Yes	Yes	18
Patient 45	1111	300	Yes	Yes	Yes	15
Patient 46	255	1068	No	Yes	Yes	12
Patient 47	746	272	Yes	Yes	Yes	Alive
Patient 48	1	300	No	Yes	Yes	5
Patient 49	189	100	Yes	Yes	Yes	Alive
Patient 50	280	300	No	Yes	Yes	10
Patient 51	217	400	No	Yes	Yes	21
Patient 52	475	1144	No	Yes	No	22
Patient 53	2342	2100	Yes	No	No	3
Patient 54	96	800	No	Yes	No	7
Patient 55	604	300	No	Yes	Yes	26
Patient 56	Unknown	300	No	Yes	Yes	16
Patient 57	190	200	No	Yes	Yes	15
Patient 58	43	900	Yes	No	No	1
Patient 59	83	506	Yes	Yes	Yes	10
Patient 60	Unknown	600	Yes	Yes	No	16
Patient 61	1662	600	Yes	No	No	Alive
Patient 62	30	900	No	Yes	Yes	18
Patient 63	63	300	Yes	Yes	Yes	11
Patient 64	470	300	Yes	Yes	Yes	27
Patient 65	2127	300	Yes	Yes	Yes	18
Patient 66	86	1664	No	Yes	Yes	16
Patient 67	9	100	No	Yes	Yes	Alive
Patient 68	297	787	No	Yes	No	Alive
Patient 69	1848	100	Yes	Yes	No	12
Patient 70	1458	300	Yes	Yes	Yes	Alive
Patient 71	Unknown	600	No	Yes	Yes	Alive
Patient 72	Unknown	100	No	Yes	Yes	10
Patient 73	180	1200	No	Yes	Yes	Alive
Patient 74	Unknown	300	Yes	Yes	Yes	19
Patient 75	189	840	No	Yes	No	21
Patient 76	143	900	Yes	Yes	Yes	Alive
Patient 77	675	300	Yes	Yes	Yes	Alive
Patient 78	877	640	Yes	Yes	Yes	10
Patient 79	624	200	Yes	Yes	Yes	18
Patient 80	Unknown	300	Yes	Yes	Yes	Alive
Patient 81	Unknown	200	Yes	Yes	Yes	11
Patient 82	879	900	Yes	Yes	Yes	Alive
Patient 83	106	300	No	Yes	No	12
Patient 84	19	300	No	Yes	Yes	2
Patient 85	58	484	No	Yes	Yes	8

Patient 86	Unknown	Unknown	Yes	Yes	Yes	13
Patient 87	69	774	No	Yes	Yes	3
Patient 88	1802	600	Yes	Yes	Yes	Alive
Patient 89	500	379	Yes	Yes	Yes	13
Patient 90	Unknown	100	No	Yes	Yes	Alive
Patient 91	2494	300	No	Yes	No	5
Patient 92	99	900	No	Yes	Yes	Alive
Patient 93	151	300	No	Yes	Yes	Alive
Patient 94	61	300	Yes	Yes	Yes	4
Patient 95	501	900	Yes	Yes	Yes	Alive
Patient 96	382	300	No	Yes	Yes	Alive
Patient 97	30	300	No	Yes	Yes	Alive
Patient 98	Unknown	Unknown	No	Yes	No	Alive
Patient 99	2015	400	Yes	No	No	3
Patient 100	165	300	Yes	Yes	No	Alive
Patient 101	53	300	No	Yes	Yes	Alive
Patient 102	50	700	No	No	Yes	Alive
Patient 103	1	600	No	Yes	Yes	Alive

Supplementary Table 3: Gabapentin treatment dosing, timing, concurrent chemotherapy/radiotherapy, and survival in GBM patients within the UCSF validation cohort.

Patient (N=36)	Dose (mg)	Daily frequency	Total daily dose (mg)	Treatment with chemoradiation	Overall survival (days)	Censored (Yes, No)
Patient 1	100	1	100	Both	1038	No
Patient 2	100	1	100	Chemotherapy only	364	No
Patient 3	100	1	100	Neither	2438	No
Patient 4	100	2	200	Both	267	No
Patient 5	100	3	300	Both	4862	Yes
Patient 6	300	1	300	Neither	1459	Yes
Patient 7	100	3	300	Both	635	No
Patient 8	300	1	300	Both	508	No
Patient 9	300	1	300	Both	598	No
Patient 10	300	1	300	Both	843	No
Patient 11	300	1	300	Both	531	No
Patient 12	300	1	300	Both	649	No
Patient 13	100	3	300	Both	1144	No
Patient 14	100	3	300	Both	977	Yes
Patient 15	100 AM, 300 PM	1, 1	400	Both	482	No
Patient 16	600	1	600	Both	4410	Yes
Patient 17	300	2	600	Radiation only	107	No
Patient 18	300	2	600	Both	969	Yes
Patient 19	300	2	600	Chemotherapy only	290	No
Patient 20	300	3	900	Both	605	No
Patient 21	300	3	900	Both	3158	No
Patient 22	300	3	900	Both	961	No
Patient 23	300	3	900	Both	708	No
Patient 24	300	3	900	Both	629	No
Patient 25	300	3	900	Neither	144	No
Patient 26	300	3	900	Both	56	No
Patient 27	300	3	900	Both	187	No
Patient 28	300	3	900	Both	673	Yes
Patient 29	300	3	900	Both	812	No
Patient 30	900	2	1800	Both	523	No
Patient 31	600	3	1800	Radiation only	353	No
Patient 32	1200	2	2400	Both	167	No
Patient 33	600	4	2400	Both	811	No
Patient 34	Unknown	Unknown	Unknown	Both	488	No
Patient 35	Unknown	Unknown	Unknown	Both	234	No
Patient 36	Unknown	Unknown	Unknown	Both	2607	No

Supplementary Table 4. Sensitivity analysis including IPTW. The results of the different Cox proportional hazards regression models are presented as unweighted and IPTW HR (95% CI) of postoperative gabapentin use compared to no postoperative gabapentin use.

**Model (independent variables)**	**IPTW HR (95%CI)**	**Unweighted HR (95%CI)**
**Main model** (age, sex, race, *MGMT* methylation, EOR, postoperative levetiracetam use, postoperative AED use, preoperative KPS)	0.64 (0.49-0.85)	0.65 (0.51-0.84)
**Main model + radiotherapy + chemotherapy**	0.71 (0.54-0.93)	0.72 (0.56-0.92)
**Main model + left frontal tumor location**	0.64 (0.49-0.85)	0.66 (0.51-0.84)

IPTW = inverse probability of treatment weighting; *MGMT* = O-6-methylguanine-DNA methyltransferase; EOR = Extent of resection; AED = Antiepileptic drug(s); KPS = Karnofsky performance status.
